# Antioxidant, Anti-Inflammatory, and Microbial-Modulating Activities of Essential Oils: Implications in Colonic Pathophysiology

**DOI:** 10.3390/ijms21114152

**Published:** 2020-06-10

**Authors:** Enzo Spisni, Giovannamaria Petrocelli, Veronica Imbesi, Renato Spigarelli, Demetrio Azzinnari, Marco Donati Sarti, Massimo Campieri, Maria Chiara Valerii

**Affiliations:** 1 Department of Biological, Geological and Environmental Sciences, University of Bologna, Via Selmi 3, 40126 Bologna, Italy; giovannam.petrocell2@unibo.it (G.P.); renato.spigarelli@studio.unibo.it (R.S.); demetrio.azzinnari1@gmail.com (D.A.); 2 Department of Medical and Surgical Sciences, University of Bologna, Via Massarenti 9, 40138 Bologna, Italy; veronica.imbesi@studio.unibo.it (V.I.); marco.donatisarti2@studio.unibo.it (M.D.S.); massimo.campieri@unibo.it (M.C.); chiaravalerii@hotmail.it (M.C.V.)

**Keywords:** essential oils, bowel, antioxidant, anti-inflammatory, microbial-modulating, colorectal cancer

## Abstract

Essential oils (EOs) are a complex mixture of hydrophobic and volatile compounds synthesized from aromatic plants, most of them commonly used in the human diet. In recent years, many studies have analyzed their antimicrobial, antioxidant, anti-inflammatory, immunomodulatory and anticancer properties in vitro and on experimentally induced animal models of colitis and colorectal cancer. However, there are still few clinical studies aimed to understand their role in the modulation of the intestinal pathophysiology. Many EOs and some of their molecules have demonstrated their efficacy in inhibiting bacterial, fungi and virus replication and in modulating the inflammatory and oxidative processes that take place in experimental colitis. In addition to this, their antitumor activity against colorectal cancer models makes them extremely interesting compounds for the modulation of the pathophysiology of the large bowel. The characterization of these EOs is made difficult by their complexity and by the different compositions present in the same oil having different geographical origins. This review tries to shift the focus from the EOs to their individual compounds, to expand their possible applications in modulating colon pathophysiology.

## 1. Introduction

Essential oils (EOs) are a complex mixture of volatile compounds that are produced by aromatic plants as secondary metabolites. They are present in all plant organs and their main role is to defend plants from aggressions by bacteria, fungi and viruses, but also by insects. There are huge amounts of EOs obtained from different plants around the world, and most of them have been at least partially characterized for their antimicrobial activity against Gram-Positive and Gram-Negative bacteria, but also against other microorganisms such as fungi and virus. The composition of the EOs was selected by nature during an evolutionary process lasting millions of years. Their activity is the result of the competitive selection process acting on their antibacterial, antifungal and antiviral activities in a continuous evolutionary conflict between the survival of plants and the microbial aggressions. The antimicrobial strategies of EOs have been to develop multi-target mechanisms of action which make it very difficult for microorganisms to become resistant to these compounds [1]. The enormous potentials for the possible use of EOs as therapeutic agents against human pathogen microorganisms have so far been little exploited, due to three major problems. The first one is that EOs are complex and variable mixtures of bioactive compounds such as terpenoids and non-terpenic molecules, which change in their relative proportions, within the same species of plant, depending on the latitude, the season, the type of soil and the agricultural conditions in which plants have grown. The second is that EOs may be toxic to humans, at doses that are not much higher than those at which they may exert their therapeutic effects. The last one is that most of their components are classified as mucosa and skin irritants and they may cause irritative or also allergic epithelial reactions. Toxicity of some EO components for oral administration are showed in Table 1. 

**Figure 1:**
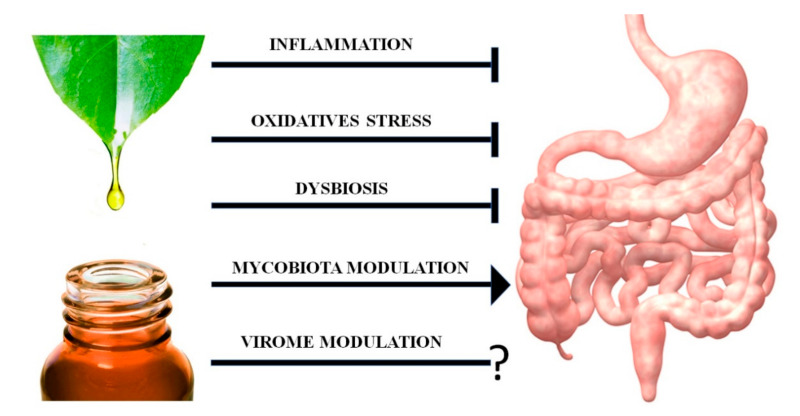
Multiple effects of essential oils, generally extracted by distillation from aromatic plants, on the gastrointestinal system. These effects have been obtained both from essential oils as they are and from their single bioactive compounds. The anti-tumor action of essential oils (EOs) is due both to the single effects shown in the figure and to specific actions directed against colorectal cancer cells.

In recent years the potential therapeutical activities of EOs and of their single components have been extended because of their emerging role as antioxidants and immunomodulatory agents (Figure 1) [2]. 

The excessive amount of reactive oxygen species (ROS) is at the basis of degenerative processes such as lipid peroxidation, protein glycation/oxidation and nitration, enzyme inactivation and DNA damages that occur in many non-communicable diseases, including colitis and neurodegenerative diseases [3,4]. Some EO single molecules, such as geraniol, easily cross the blood–brain barrier and then reach all the organs in which they can exert their antioxidant activities [5]. ROS exert multiple modulating effects on inflammation and play a key role in the regulation of immune responses [6]. EOs and their molecules are capable of modulating different signaling pathways that are overactivated or downregulated during acute or chronic inflammation responses [7].

The recent discovery of the complexity of the human intestinal microbiota, composed of bacteria, fungi and viruses, and its intricate pathophysiological relationships with the immune system and the enteric nervous system, makes EOs truly interesting for their antimicrobial activities, often selective for the different microbial components. From this point of view, EOs could be considered potential powerful modulators of the intestinal microbiota. Unfortunately, most EO molecules are quickly assimilated into the small intestine and do not reach the colon, where most of the intestinal microbiota is known to reside. The release of EOs into the colon, therefore, becomes a fundamental point to allow these compounds to effectively modulate the pathophysiology of the colon. From this point of view, our research group is one of the first in the world to have used a single EO molecule in human clinical trials, to modulate the microbiota and manage the symptoms associated to a functional bowel disorder such as the irritable bowel syndrome (IBS) [8].

This review explores, in a broad and modern way, the knowledge that can lead to the effective use of EOs and/or their single molecules in the modulation of the physiology and pathology of the large bowel. The major components of the most common essential oils are shown in Table 2, together with their chemical structure and major biological activity.

## 2. EOs as Intestinal Anti-Inflammatory Agents

Several studies have been conducted to evaluate the effects of EOs in colon inflammation [9,10,11,12,13,14,15,16,17,18]. In particular, several preclinical studies have been conducted on validated models of colitis induced in rats or mice by administration of Dextran Sulphate Sodium (DSS), Trinitrobenzenesulfonic acid (TNBS) or Acetic Acid. While DSS and Acetic Acid induces chemical damage to the epithelial cells, with consequent disruption of mucosal barrier and activation of immune cells in the lamina propria by the intestinal microbiota, TNBS acts by directly haptenizing colonic autologous/microbial proteins [9,10,11,12,13,14,15,16,17,18]. From this point of view, DSS and Acetic Acid are models used in a translational way for ulcerative colitis studies, while TNBS is a model with some characteristics that bring it closer to Crohn’s disease. These models allow researchers to evaluate the effects of different EOs on clinical, histological, serological and molecular markers of colitis. Clinical features of the experimental colitis are mainly represented by weight loss, changes in stool consistency and rectal bleeding, usually scored to obtain a Disease Activity Index (DAI). Histological analyses are performed to evaluate the extent of ulcerated areas, alterations of mucosal architecture and inflammatory cell infiltrations. The molecular markers that are more often evaluated comprise some circulating pro-inflammatory cytokines and the colonic expression of a plethora of enzymes and factors involved in inflammation. They include Cyclooxygenase-2 (COX-2), Nitric Oxid Synthase (iNOS), peroxisome proliferator-activated receptor-gamma (PPARγ), Nuclear Factor kappa-light-chain-enhancer of activated B cells (NF-kβ) or Myeloperoxydase (MPO) [9]. The anti-inflammatory effects of EOs on colitis models are summarized in Table 3.

**Figure 2:**
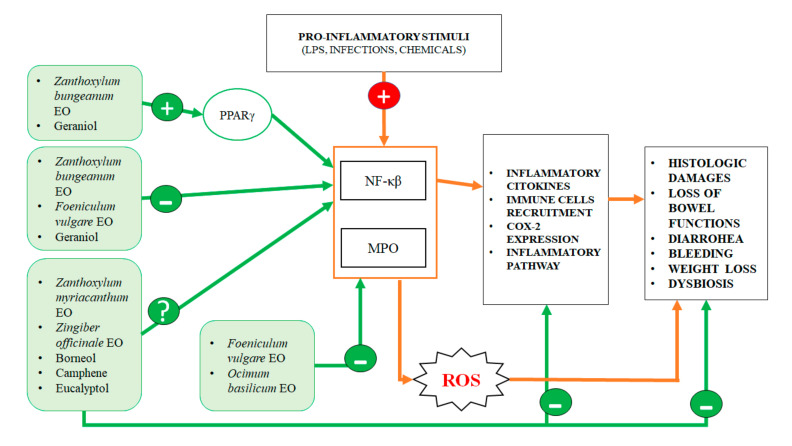
Intestinal anti-inflammatory effects of essential oils (EOs). Pro-inflammatory stimuli, such as chemicals (Dextran Sulphate Sodium (DSS), Acetic Acid, Trinitrobenzenesulfonic acid (TNBS)), bacterial toxins (such as Lipopolysaccharide (LPS)) or pathobionts infections increase the inflammatory response into the gut, with increased expression of Nuclear Factor kappa-light-chain-enhancer of activated B cells (NF-kβ) or Myeloperoxydase (MPO) enzyme. Recruitment and activation of immune cells increase cellular and histological damages that mainly involve the large bowel. EOs and their single components act at multiple levels, counteracting inflammation and consequently decreasing damages to the intestinal mucosa and to the intestinal wall. EO, essential oil; PPARγ, peroxisome proliferator-activated receptor-gamma; COX-2, Cyclooxygenase-2; ROS, reactive oxygen species.

With regard to the mechanisms of action of EOs and their single molecules at the cellular and molecular levels, we underline that it is almost impossible to understand which molecule may exert anti-inflammatory effects when EOs are used. Even in studies carried out on single EO molecules, it is not easy to define a main molecular target, because these compounds act in a multitarget manner (Figure 2). For example, the anti-inflammatory effect of these molecules could be linked to direct interaction with pro-inflammatory proteins, such as NF-kβ, PPARγ COX-2 and iNOS, but this direct interaction still remains to be demonstrated for the majority of these molecules. A specific interaction between borneol, camphene and eucalyptol, major components of thyme EO, and pro-inflammatory enzymes such as iNOS and COX-2 has been only indirectly demonstrated in vitro [18]. The multitarget effect of geraniol has been clearly demonstrated in vitro, since it is capable of regulating Wnt/β-catenin, p38MAPK, NFκB, PPARγ and COX-2 signaling pathways. The transcription factor NF-kβ seems to be a common target for many different EO molecules, such as geraniol, eucalyptol, α-pinene, and camphor [19], even if a definitive demonstration of the binding between these molecules and their supposed target is still lacking and should be provided by using molecular modeling studies. 

Conceptually, however, these studies on colitis should be divided between those that delivered EO as it is, without carrier or controlled release and those in which the EO was delivered with retarded release systems, in order to really reach the colon. Only by using these controlled release systems can the effects of these EOs be analyzed at the colon level, where the experimentally-induced inflammation mainly occurs. Basing on available studies, we can conclude that EOs and their constituents could be very effective against the inflammatory component of experimental colitis. Unfortunately, EO complexity makes it difficult to identify all the possible molecular targets responsible for these strong anti-inflammatory effects.

## 3. Antioxidant Effect of EO Components into the Gut

The majority of studies on the antioxidant effects of EOs, available in the scientific literature, are based on in vitro approaches. A limiting factor of these studies is that EOs can themselves be oxidized within the intestinal lumen or into the stomach, thus losing some of their antioxidant properties even before reaching the small intestine.

Geraniol showed good antioxidant proprieties in vitro: palmrose and citronella EOs, mainly composed of geraniol, have demonstrated to have an effective antioxidant activity in vitro on human lymphocyte cells. In this model, geraniol-containing EOs, at a relatively low concentration (125 ppm), protected lymphocytes from DNA methylation damages induced by methyl methanesulfonate [20]. These serum doses of geraniol are easy to achieve with a diet rich in aromatic plants or with food supplements [21]. Recent studies have shown that geraniol administration reduced the intestinal inflammation induced by DSS [7], but these anti-inflammatory effects could be also linked to its antioxidant activity, since its administration resulted in a decreased iNOS activity and a decreased lipid peroxidation, in a rat model of colitis [22]. Geraniol seems to exert its antioxidant activity also indirectly, by increasing the synthesis of liver antioxidant enzymes after oral administration at 120 mg/kg, in mice [7].

Ginger EO administration at 200, and 400 mg/kg, significantly reduced the intestinal lipid peroxidation, increased the expression of intestinal antioxidant enzymes and serum glutathione level in a model of colitis induced by acetic acid in rats. In this model, ginger EO was able to induce free radicals neutralization and to protect the cell membrane lipids from oxidation, in a dose-dependent manner [14]. A recent study has evaluated antioxidant proprieties of carvacrol (5-isopropyl-2-methylphenol) which is a major monoterpenic component of origan (*Oreganum vulgarea*), a plant widely used in Mediterranean cuisine. Carvacrol was used in a model of experimental induction of colorectal carcinoma, by using 1,2 dimethylhydrazine and DSS, in rats. Carvacrol was orally administrated before and after tumor induction at a dosage of 50 mg/kg. Results of this and other studies showed that carvacrol was an excellent antioxidant agent and reduced colonocyte damages caused by ROS [23,24]. Thymol is a natural phenol monoterpene isomer of carvacrol. Thymol is one of the major constituents (20–65%) of thyme (*Thymus vulgaris*) EO. An in vitro study on colon epithelial cells showed that thymol, at low doses (12.5 ppm), was a protective compound against oxidative DNA damage [21]. 

Twelve aromatic molecules from basil and thyme EOs have been analyzed for their antioxidant activities, and in particular eugenol, 4-allylphenol, thymol and carvacrol (5 µg/mL) have shown greater antioxidant activities, measured in vitro on by using the aldehyde/carboxylic acid assay [23]. Basil EO, orally-administered, has shown beneficial effects in a model of colitis induced by acetic acid in rats, at two different doses of 160 mg/kg and 320 mg/kg die. These effects were also linked to the reduction of MPO activity in the colon wall, an enzyme clearly involved in the oxidative damages induced by colitis [15]. 

**Figure 3:**
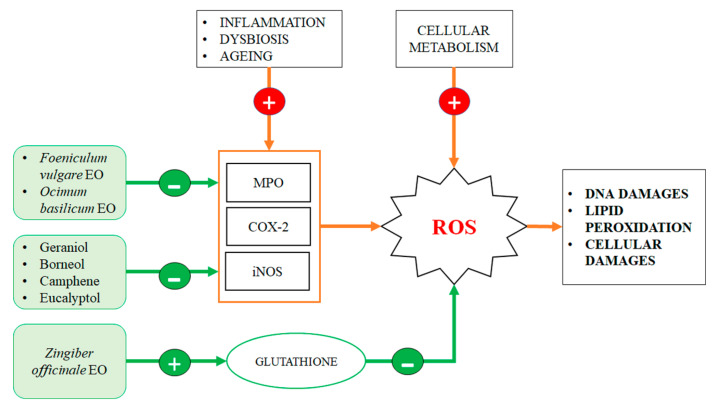
Antioxidant effect of essential olis (EOs) in the gut. The chronic low-grade inflammation or dysbiosis that very soon occurs into the gut and in the gut wall increases the level of reactive oxygen species (ROS). Their increased levels are effectively counteracted by EOs that are able to reduce the activity and expression of enzymes, such as Myeloperoxydase (MPO), Cyclooxygenase-2 (COX-2) or inducible Nitric oxide synthase(iNOS), which are the ones most responsible for ROS production and for the oxidative damages related to them.

Despite this, further confirmations should be provided by human clinical trials. Basing on available data we can confirm that EOs and their constituents have interesting antioxidant properties that could justify their use as therapeutic agents against chronic intestinal oxidative damages (Figure 3). 

## 4. Intestinal Microbiota Modulation Exerted by EO Components

### 4.1. Antibacterial Proprieties of EOs and Bacterial Microbiota Modulation 

There are approximately 100 trillion cells in the human body, and more than 90% of them are microbes. They make up the human microbiota, consisting of bacteria, fungi and even viruses, mainly located in the intestine where they form the intestinal microbiome. Microbiota can be considered a complex human organ which closely interacts with the Gut-Associated Linfoid Tissue (GALT) and with the enteric nervous system. It is involved in many digestive functions, but it is also able to modulate the physiology of the immune system both locally and in the whole body. Quantitative and qualitative microbiota alterations, known as dysbiosis, may be involved in the development or in the chronicization of several diseases [24]. The analysis of the bacterial component of the intestinal microbiota, thought their 16S rRNA sequences, allowed to identify 4 major phyla, Firmicutes (79%), Bacteroidetes (17%), Actinobacteria (3%) and Proteobacteria (1%). At a lower taxonomic level, the most represented bacterial genera were found to be Faecalibacterium, Ruminococcus, Eubacterium, Dorea, Bacteroides, Alistipes and Bifidobacterium [25]. The microbiota normally represents an ecologically stable environment, but pathogenic bacterial strands or xenobiotics can interfere in this equilibrium and give rise to dysbiosis and/or colitis. The use of broad-spectrum antibiotics to counteract infectious diseases is often associated with the onset of antibiotic resistance phenomena, other than cause a transient dysbiosis in the gastrointestinal tract. In the last few years, a great effort has been made to find new strategies to overcome the rising issue of antibiotic-resistance. In this scenario, EOs may have a consistent role thanks to their capacity to control and modulate bacterial growth, acting both as bacteriostatic or bactericidal agents [26]. In fact, due to their lipophilic properties, EOs can penetrate membranes, and damage bacterial cell structure, making their membranes more unstable and permeable. Membrane disruption may also lead to bacterial death caused by the significant leak of ions and other essential cytosolic components. These EO effects are generally more pronounced on Gram-Positive bacteria with respect to Gram-Negative ones [27]. However, it has been demonstrated that EOs can also affect the bacterial cell-wall and restore antibiotic susceptibility in drug-resistant Gram-negative bacterial strains [28].

Several studies have explored the antibacterial properties of EO single molecules. Among them, the most studied was certainly geraniol, for its interesting antimicrobial potential. Geraniol antibacterial activity seems to be linked to his property to destabilize bacterial cell wall and damage transmembrane efflux pumps, thus restoring drug-sensitivity in different bacterial antibiotic-resistant strains, such as *Enterobacter aerogenes*, *Escherichia coli*, *Pseudomonas aeruginosa* and *Acinetobacter baumannii* [29]. Despite being absorbed very quickly and in an active manner by the small intestine mucosa, geraniol is reported to positively modulate the colitis-associated dysbiosis when administered by oral route by using a controlled delivery system based on microencapsulation [7]. In mice but also in humans, geraniol has demonstrated to act as an excellent modulator of intestinal microbiota, capable to boost populations of butyrate-producer bacteria such as Collinsella and Faecalibacterium, normally reduced in the dysbiotic human intestinal flora of IBS patients [8]. It is interesting to note how geraniol antibacterial activities were selective for pathogenic bacteria and do not involved commensal species [30]. For these reasons, geraniol can be considered as an efficient positive modulator of the intestinal microbiota.

Another interesting EO molecule with antibacterial activities is eugenol (2-Methoxy-4-(prop-2-en-1-yl)-phenol), the major compound present in clove oil, but also found in many other EOs. Eugenol has demonstrated antimicrobial activities based on a non-specific permeabilization of the bacterial membrane with depletion of cytosolic molecules such as adenosin triphosphate (ATP), necessary for bacterial metabolism and survival [31]. This effect has been observed against *E. coli*, *Listeria monocytogenes* and *Lactobacillus sakei* using the relatively low concentration of 10 mM [32]. Eugenol antibacterial effects against the pathogen *L. monocytogenes* have been analyzed in-depth and the principal mechanism of action identified was the alteration of the respiratory bacterial chain associated with DNA damages [32]. In mice, orally administrated eugenol improved the secretion of the intestinal mucus, creating a thicker intestinal layer associated with positive changes of the mucosal microbiota ecology. In particular, it has been shown that eugenol inhibited the intestinal adherence of *Citrobacter rodentium*, a mice pathogen that shares several biochemical features with *Clostridium difficile* in humans [33]. Another interesting antimicrobial effect of eugenol was observed in *P. aeruginosa* and *E. coli*, where this compound was able to inhibit their chemical communication system, also known as quorum sensing [34]. 

Cinnamaldehyde (2E-3-Phenylprop-2-enal) is a phenylpropanoid naturally present in the plant of the genus Cinnamon. Cinnamaldehyde is one of the most studied EO molecules and it has been already approved as antimicrobial food preservative [2]. Antibacterial effects of cinnamaldehyde have been demonstrated by using many different bacterial models, but only a few studies evaluated its impact on the whole intestinal microbiota. In vitro, cinnamaldehyde was capable to inhibit the growth of potentially pathogenic bacteria such as *Staphylococcus aureus*, *Enterobacter cloacae*, *A. baumannii* and *L. monocytogenes* [35] and it was able to kill a pathogenic strand of *E. coli* at a very low concentration (0.05% *v*/*v*) [36]. One of the proposed antibacterial mechanisms of cinnamaldehyde inhibition of *E. coli* growth was the inactivation of its acetyl-CoA carboxylase enzyme [37]. Other studies showed that cinnamaldehyde antimicrobial activity has a broad spectrum of action, being effective also against *Enterococcus faecalis, Enterococcus faecium, E. aerogenes Salmonella enterica* and *Clostridium perfringens* [38]. Finally, with a concentration of 20 µg/mL, cinnamaldehyde was also capable to improve the bactericidal efficacy of the antibiotic clindamycin on *C. difficile*, significantly decreasing its minimum inhibitory concentration (MIC) from 4.0 to 0.25 µg/mL [39]. In vivo, only a few studies have been conducted on cinnamaldehyde, perhaps because of its strong aggressiveness towards the mucosal epithelia. Nevertheless, in animal experimental colitis, the oral administration of cinnamon EO (approx. 70% in cinnamaldehyde) at 10 mg/kg or 15 mg/kg lead to an improvement of the ecological biodiversity of the intestinal microbiota. Short-chain fatty acids (SCFA)-producing bacteria family, such as Bacteroidaceae, were increased while intestinal Helicobacter and Bacteroides were reduced [40]. In broiler and duck farming, the supplementation of food with a mixture of thymol and cinnamaldehyde improved animal growth performances and positively modulated their intestinal microbiota composition, boosting healthy bacteria and reducing anaerobic coliforms and lactose-negative enterobacteria [41,42].

Thymol is effective at extremely low concentrations (as low as 300 ppm) against the growth of many species of pathogenic bacteria that colonize the human intestine, such as *C. difficile*, *C. perfringens*, *Propionibacterium shermanii*, *Propionibacterium freudenreichii* and *Bacteroides thetaiotaomicron*. The negative aspect of its antimicrobial activity is that thymol was not selective, and could also have a negative impact on commensal bacteria [30]. On the other hand, EOs in which thymol is a major component have clearly shown to have a wide-spectrum bactericide effects on different pathogenic species such as *L. monocytogenes*, *E. coli*, *S. enterica*, *S. aureus*, *Clostridium botulinum*, *C. perfringens*, *Shigella sonnei*, *Sarcina lutea*, *Mariniluteicoccus flavus*, *Brochothrix thermosphacta*, *Listeria innocua*, *L. monocytogenes*, *Pseudomonas putida* and *Shewanella putrefaciens* [26]. Moreover, thymol seems to be effective also against the bacterial biofilm formed by β-lactamase-producing enteric bacteria [43]. It is still debated if thymol could be or not degraded by the intestinal microbiome since it was found to be not effective against fecal fermentation reactions [44]. Nevertheless, in weaning piglets, thymol associated with carvacrol was capable of positively modulating their microbiota by increasing populations of the Lactobacillus genus and by reducing populations of Enterococcus and Escherichia genera [45].

In vitro, carvacrol was showed to inhibit bacterial adhesion, invasion and biofilm development in cultured intestinal cells [46]. In the farmed broiler, treatment with carvacrol-rich EOs was tested to control the pathogenic bacteria spreading inside the farms. The results of these studies demonstrated that carvacrol reduced the microbial counts of *E. coli* and different *Salmonella* species in the small intestine of farmed chicken [46]. Moreover, carvacrol administration to broiler chickens was capable of eliminating the intestinal presence of *Campylobacter spp*. after 21 days of daily oral administration at 120–300 mg/kg. This effect was probably linked to the enhanced growth of bacteria of the *Lactobacillus* genus, that were found to be increased in chicken microbiota, after carvacrol administration. *E. coli* growth in the cecum of chickens was found to be significantly reduced by carvacrol. For these reasons, this molecule is today the most used in organic breeding to positively modulate gut microbiota and improve the growth performance of farmed chickens [47]. In intensive breeding practices carvacrol is often associated with thymol, since there is strong evidence that the combination of the two was more effective in decreasing intestinal pathogens and increasing the growth performance of chickens [48,49].

Limonene (1-Methyl-4-(prop-1-en-2-yl)-cyclohex-1-ene) is a cyclic monoterpene present in a high amount in EO of citrus fruit peels and, in smaller concentrations, in many other aromatic plant EOs. Limonene has widely demonstrated antimicrobial and anti-inflammatory effects in vivo. In mice, daily oral administration of 160 mg (8000 mg/kg) of orange oil, rich in limonene, modulates the mice microbiota by enhancing the relative abundance of the Lactobacillus genus and reducing the presence of SCFA-producing bacteria [50]. Its particular ability to reduce the SCFA synthesis has been exploited in a mouse model of obesity to reduce weight gain. Mice fed with a high-fat diet (HFD) were treated daily with microencapsulated sweet orange oil for 15 days (2 mL of suspension of microcapsules containing 18 g/L of sweet orange EO rich in limonene). The result of this study showed a reduced body weight in treated mice, associated with a modulation of the intestinal microbiota. Specifically, the Bifidobacterium population was enhanced with an overall reduction of the intestinal chronic inflammation induced by the HFD, in treated mice [51]. Despite the low toxicity of limonene, which would not rise concerns, it should be noted that these effects on the microbiota were obtained only with high dosages of this compound.

Eucalyptol (1,3,3-Trimethyl-2-oxabicyclo[2.2.2]octane) is a cyclic ether and a monoterpenoid. It is the major compound in *Eucalyptus* EO, but it can be also found in many other officinal plants EOs. A recent review indicates that *Eucalyptus* EO has extraordinary antimicrobial activities. Eucalyptus EO has shown to be effective against a plethora of bacteria species and among them *S. aureus*, *E. coli*, *Bacillus subtilis*, *Klebsiella pneumonia*, *Salmonella enteritidis* and *P. aeruginosa* [52]. Nevertheless, *Eucalyptus* EO also contains high amounts of other antimicrobial components besides eucalyptol, therefore not all of *Eucalyptus* EO antibacterial activity can be ascribed to the presence of eucalyptol. However, literature data regarding eucalyptol antimicrobial activity are very limited, and new studies focused on this interesting compound are needed.

Menthol (5-Methyl-2-(propan-2-yl)cyclohexan-1-ol) is a chiral alcohol and the main molecule present in Cornmint and Peppermint EOs. It has been well known for its use in foods as a cooling and minty-smell aroma. Many in vitro studies, reviewed by Kamatou and coworkers [53] focused on its antibacterial activities. Interestingly, in vitro, menthol was capable to drastically decrease *C. difficile* viability at 18.8 mg/mL, with a dose-dependent effect. Its mechanism of action seems to be due to the significant leakage of cellular ATP induced by menthol in this pathogenic bacterium [54].

### 4.2. EOs in the Modulation of Intestinal Mycobiome

Fungi were reported to represent about 0.1% of all the microorganisms present in the gastrointestinal tracts. Maybe also for this reason, despite the presence of fungi in the intestine has been known for many years, in depth studies of the human mycobiome were only recently performed [55].

Humans are hosts to a wide number of fungi species that coexist with the other microorganisms into the gut in a complex ecological relationship of interdependence [56]. Together with bacteria, fungi contribute to the modulation of the intestinal immune system [57]. Many of them have a clear pathogenic potential even if, physiologically, they are commensals in our bodies. Only in some specific conditions their overgrowth can lead to well-known mycosis. The best known fungal pathogen of humans is certainly *Candida albicans,* which is a normal component of the gut mycobiota but may causes candidiasis in case of its intestinal and vaginal overgrowth [58]. An altered intestinal mycobiota has also been observed in other human pathological conditions, such as IBS [59], inflammatory bowel disease (IBD) [60] and also autism-spectrum disorders and Rett syndrome [61]. Since human mycobiome is altered in some diseases, perhaps contributing to their pathogenesis, the therapeutic manipulation of the mycobiome could be a useful approach to treat and/or prevent these diseases [62]. EOs antimycotic activities are characterized by a broad spectrum of actions [63]. *C. albicans* is responsible for the majority of fungal infections in humans and is certainly the most studied mycobiota pathogen. The overgrowth of *C. albicans* is usually controlled by the immune system of the host; however, in particular conditions such as in immunocompromised patients, this microorganism may cause severe infections [64]. For this reason, *C. albicans* is one of the main target for studies focusing the antifungal effect of EOs and their single molecules. The antifungal activities of EO obtained from *Thymus vulgaris*, *Citrus limonum*, *Pelargonium graveolens*, *Cinnamomum cassia*, *Ocimum basilicum*, and *Eugenia caryophyllus* have been evaluated against clinical isolates of *C. albicans* and *Candida glabrata*. All of these EOs exhibited both fungistatic and fungicidal activity toward these two Candida species, but cinnamon oil demonstrated the highest activity [64]. Since the most represented active compounds of Cinnamomum EO is cinnamaldehyde, many studies have been addressed to analyse in depth its activity against *C. albicans.* The MIC_90_ at which cinnamaldehyde has been shown to inhibit *C. albicans* growht ranged from 125 to 450 µg/mL [65].

Limonene has shown to possess strong antifungal properties [66] and in particular an excellent anti-Candida activity. A recent study analyzed the efficacy of this compound against the growth of *C. albicans* isolates, including fuconazole-resistant strains. In this study, the in vitro growth of 35 clinical isolates of *C. albicans* were completely inhibited at doses ranging between 5 mM and 20 mM of limonene. Furthermore, limonene inhibited the adhesion, development and maturation of the *C. albicans* biofilm with 50% of inhibition occurred at doses between 1.8 and 7.4 mM. At the dose of 20 mM, limonene was also capable to degrade 70% of mature biofilm [67].

Mentha EOs obtained from *M. piperita*, *M. spicata*, *M. longifolia*, *M. pulegium*, *M. cervina,* and *M. suaveolens,* have demonstrated good antimycotic activities against different fungi genera, including Candida [68]. Menthol and (+)-carvone are the major components of peppermint EOs and both exhibited strong antifungal activity in vitro. These activities were evident only at a relatively high doses: peppermint oil inhibited the in vitro growth of *C. albicans* at 20 mg/mL in agar dishes, whereas caraway oil showed inhibitory effects at lower doses, in the range 5–10 mg/mL [69]. A patented peppermint and caraway oils formulation, called Menthacarin^®^, was tested in an IBS animal model, showing to be effective in alleviating abdominal pain in rats via mycobiome modulation, suggesting a possible role of mycobiota dysbiosis in the etiopathogenesis of IBS [70].

*Thymus vulgaris* EO has also shown to be effective against fungi pathobionts capable to infect humans. A study on Dermatophyte, fungi that can cause superficial infections of the skin, and on Aspergillus, fungus genera that can cause respiratory infections, reported MIC values for *Thymus vulgaris* EO ranging from 0.16 to 0.32 µL/mL. Higher MIC values, between 0.32 and 0.64 µL/mL, were reported for *Candida* spp. The antifungal activity of this EO has been attributed to its two major components: thymol and carvacrol, that accounted respectively 26% and 21% of *Thymus vulgaris* EO [70]. Both these phenolic compounds seem to act by disrupting the fungal cell membranes [27]. An interesting study evaluated the antifungal activity of thymol in comparison with miconazole, a classical antifungal medication, against *C. albicans* growth and biofilms formation. The results of this study demonstrated that these two molecules were equally effective against *C. albicans* with no statistically difference between the two treatments, confirming an extraordinary antimycotic effect of thymol. However, relatively higher concentration was necessary for thymol, with a MIC that corresponded to 350 µL/mL vs. 75.15 µL/mL for miconazole [71]. Thymol antifungal activity was tested against other Candida species, and it showed to be effective also against *C. tropicalis.* [72].

Clove EO has been traditionally used in dentistry for its anesthetic and antimicrobial activities [73]. Its anti-fungal action has been attributed to eugenol, the major clove oil molecule. A recent study indicated that Clove EO, at concentrations that ranged between 0.03% and 0.25% (*v*/*v*), inhibited the biofilm formation in many Candida species, grown on different substrates [74]. For what the mechanism of action concerns, eugenol was able to cause permanent injury to the cell membranes of *C. albicans* and morphological alterations to its cell wall [27,75]. The activity of eugenol against *C. albicans* was multitarget and also targeted enzymatic pathways, such as the H^+^-ATPase in mitochondria [76]. Eugenol treatment also induced an overall oxidative damage to the fungal cell (lipid peroxidation), and these multiple damages may finally lead to cell death [77].

### 4.3. EO Antiviral Activity and Their Possible Effects on the Intestinal Virome

The antiviral activity of EOs has been established for different viruses, and it can be directed against the viral particle or against their intracellular replication process [78]. Several studies demonstrated that the Herpes simplex viruses, type 1 and 2 (HSV-1 and HSV-2), Influenza A virus and Coronavirus may be sensitive to EOs and to their single molecules [79,80,81]. It is interesting to observe that many HSV strains, resistant to synthetic antiviral drugs, are instead sensible to EOs and to their components. That is probably because they have a multitarget mechanism of action compared to specific drugs that usually target single virus component or single metabolic pathways. For this reason, it is reasonable to expect that antiviral drugs and EOs could act in synergy [78].

Bovine viral diarrhea virus (BVDV) is considered a good animal model for the human hepatitis C virus. This virus has been successfully treated with Basil EO and single monoterpenes that are major components of this EO: camphor, thymol and eucalyptol. To perform the tests, Basil EO and monoterpenes were used at a concentration of 64 mg/mL. Results obtained in this in vitro study showed that while the Basil EO did not demonstrated to have significant antiviral activities, its single monoterpenes significantly decreased BVDV infectivity on bovine kidney cells, acting directly on the viral particle [82]. These results are interesting because they suggest that for specific antimicrobial applications, it is much more useful to use individual components of EOs rather than EOs as such. Since the antimicrobial and antiviral activity of single EO molecules are multitarget activities, it is very important to design new study focused on single EO constituents and not on blends of different EOs, that are very difficult to analyze and to replicate. Antiviral properties of carvacrol have been demonstrated on nonenveloped murine norovirus (MNV), a good model for the human norovirus. Carvacrol 0.25% and 0.5% (*v*/*v*) reduced virus propagation on a murine monocyte cell line, demonstrating its ability to inactivate MNV acting firstly on the viral capsid and afterwards directly on its RNA. This underlines the efficacy of carvacrol as a natural surface disinfectant and as a food preservative to control human norovirus, which are the most frequent cause of food-borne viral diseases in humans, causing non-bacterial gastroenteritis. [83].

To date, no study has been performed to understand the impact of EOs on the intestinal virome. The main physiological viral component of the gastrointestinal tract is represented by prophages or phages [84]. The bacteriophage component is mainly composed by temperate virus of the Caudovirales order, but most of the detected viral sequences in human gut virome could not be attributed to known viruses [85] and to date it is estimated that the number of viruses in human stools is up to 10^9^ per gram [86]. Despite this, it is clear that EOs may impact the intestinal virome composition by modulating all the microbiota components, it could be really difficult to understand the direct impact of EOs on the intestinal viruses and the consequences of this modulation on the intestinal ecology.

To date, there is some evidence that EOs may be effective against different virus replication, but there are still not enough data to predict what could be the impact of EOs on viral infectious diseases of the gastrointestinal tract. The presence of a very complex microenvironment makes it difficult to understand if EOs can really act against the pathogen virus particles or if they reach mucosa cells only after the infection. Since EOs are potentially able to act both on bacteria and viruses, in pathological conditions it could be difficult to understand the real targets of these compounds. For example, it has been demonstrated that Norovirus persistent infection can be sustained by gut bacteria dysbiosis, and the use of antibiotics in infected mice is able to counteract the viral replication [87,88].

## 5. Essential Oils Component with Antitumor Activity in Colorectal Carcinogenesis

Nowadays, colorectal cancer (CRC) is one of the most common tumours worldwide. It represents the second cause of cancer death in Europe, even if mortality is decreasing due to the new screening programs and improvement in therapies [89]. The aetiology of CRC is not only related to genetic and environmental factors but also to gut microbiota and chronic colonic inflammation. Genetic factors include mutations in genes regulating enterocyte cell growth, proliferation, differentiation or cell cycle control or polymorphisms of several proteins involved in DNA repair and transcription [90,91,92,93]. Among the environmental factors, the most important seems to be the high consumption of red meat, smoking and drinking alcohol in huge amounts [94,95]. The involvement of the gut microbiota in CRC is related to the production of noxious metabolites by bacteria, such as secondary bile acids, polyamines or genotoxins [96]. These metabolites may cause to colonocytes oxidative stress, direct DNA damage or induce inflammation [97]. Chronic inflammation condition, such as those linked to IBD, are also recognised as a risk factors for CRC development [98]. CRC manifestation can be sporadic or have a familial predisposition, that’s the case of familial adenomatous polyposis (FAP) and other syndromes like Peutz–Jeghers, serrated polyposis and Lynch [99,100]. Classical CRC therapies include surgical treatments, radiotherapy or chemotherapy [101], which are associated with important side effects and with the development of drug resistance [102,103]. Recently new pharmacological approaches have been successfully developed for CRC, including biological drugs, aimed at the treatment of previously diagnosed cancers [104]. Despite the presence of different therapeutic strategies for CRC, we must not forget the potential of natural anticancer substances, especially in the prevention of a neoplasia which requires long times to transform from a benign dysplasia to a malignant adenocarcinoma. Several EOs and their single components have been tested in vitro and/or in vivo by using CRC models and have been proved to be valid antitumoral molecules for this cancer. Carvacrol was tested on different CRC cell lines (HTC-116 and LoVo), where it was determined the reduction of proliferation and cell cycle arrest in G2/M phase, associated with the reduction of cellular invasion and migration proprieties [105]. Geraniol has shown a strong cytotoxic effect on Colo-205 cell line [106] but it was not able to induce apoptosis on Caco-2 cell line, where it only showed a cytostatic effect, by arresting their cell cycle in S phase [107]. This demonstrate how the same compound may not have the same effect on different cellular CRC models. Geraniol also demonstrated its anticancer property against the regulation of polyamine metabolism, that is another target of cancer therapies [107,108]. In different CRC cell lines, Geraniol has been shown to downregulate the ornithine decarboxylase (ODC) and to upregulate S-Adenosylmethionine decarboxylase (AdoMetDC), two enzymes involved in polyamine catabolism and elimination [107]. Thymol has shown cytotoxic effect against HTC-116 cell line by inducing ROS production and DNA damages. It also induced cell-death by affecting cancer cell mitochondrial pathways [109]. On the other hand, thymol, geraniol, nerolidol, and methyleugenol, at low doses, have demonstrated to have genoprotective effects against oxidative and DNA methylation damages in HT-29 cell line [21]. These data underline the importance of the effective dosage of these substances that reaches the colon, since all of them are subject to intestinal absorption.

Cinnamaldehyde have been tested on several CRC cell lines and, like others EO components, it caused the inhibition of proliferation and the induction of apoptosis (by PI3K/Akt inhibition and by increasing Bax/Bcl-2 ratio) associated with a reduction of invasion and migration capability of SW-480, HCT-116 and LoVo cells (by increasing E-cadherin levels and downregulating MMP-2 and MMP-9 enzymes) [110]. For these reasons, cinnamaldehyde has been used for the development of an aspirin-like drug for the prevention of CRC [111]. Cinnamaldehyde has also been associated with camptothecin, a hydrophobic anticancer drug, and then incorporated in polymeric micelles to obtain a controlled release system based on pH-gradients. These micelles have shown to induce apoptosis and generate intracellular ROS with synergistic anticancer effects between cinnamaldehyde and camptothecin both on in vitro and in vivo models of SW-620 human colon tumour cell or bearing mice [112]. So, the anticancer activities of EOs compounds can reduce drug resistance and sensitize cancer cells to traditional chemotherapeutic agents, by acting synergistically with them. Geraniol has been reported to sensitize Caco-2 cell line to 5-Fluorouracil (5-FU), by altering cell membrane potentials and facilitating the uptake of 5-FU [113,114]. The synergistic effect of Geraniol and 5-FU has been investigated both in vitro and in vivo. Geraniol potentiates 5-FU growth inhibition activity on SW-620 and Caco-2 cell lines by downregulating the thymidylate synthase and thymidine kinase, two enzymes related to 5-FU cytotoxicity. These data have been confirmed in vivo on nude mice grafted with the human colorectal cells TC-118 [115]. In a similar manner thymoquinone, a compound of *Nigella sativa* EO, demonstrated—in association with doxorubicin—an improvement of drug-antineoplastic activities on HT-29 cell line [116]. Thymoquinone also sensitized Colo-205 and HTC-116 cancer cells to cisplatin and increased cancer cell death by suppressing NF-kβ [117]. Finally, it has been shown that thymoquinone was capable to sensitize resistant LoVo colon cancer cells to the drug irinotecan by affecting ERK1/2 pathway and increasing the membrane permeability and the autophagy process [118,119].

β-caryophyllene is a natural bicyclic sesquiterpene found in many EOs, particularly in clove oil. It has been studied in association with paclitaxel and doxorubicin on DLD-1 and Caco-2 cell lines, respectively. It facilitated the passage of paclitaxel through the plasma membrane, increasing its anticancer activity [120]. Moreover, β-caryophyllene treatment induced an intracellular accumulation of doxorubicin that increased its anticancer activity [121]. β-caryophyllene has also been involved in the regulation of glucose homeostasis in CRC cells, regulating genes involved in glycolysis and cell growth and finally leading to cell growth suppression and apoptosis [122].

Taken together, these results suggest that there is a real wide possibility of using EOs or their individual compounds in CRC prevention, but also in reducing the doses of classic chemotherapy drugs adopted to treat CRC patients. This would consequently reduce the side effects of chemotherapy with a real benefit for patients during anticancer therapy but without decreasing its therapeutic efficacy.

Considering that the development of CRC can be determined by the presence of colonic chronic inflammation for a long time, the efficacy of these natural compounds is certainly maximum in terms of CRC prevention, in all subjects at high risk of developing this cancer, both for familiar history or for pre-disposing pathologies, such as IBD or FAP. In this context and within long-term therapeutic preventive strategies, it has to be stressed EOs multi-target properties, that makes the development of resistant tumour cells very difficult.

## 6. Discussion and Conclusions

EOs, but especially their single molecules, are effective multitarget modulators of the intestinal physiology and pathology. Their use to date has been limited due to several factors that include testing complex mixtures that did not allow identifying the individual active components, the difficulty of releasing these compounds in the affected intestinal tract and their toxicity and aggression on the mucous membranes which complicates their in vivo administration. Most EOs have very strong, sometimes unpleasant flavors, and in several cases they are also aggressive towards the oral, esophageal and gastric mucosa. Administration in controlled release formulations therefore often becomes a necessity. By using normal pharmacological forms such as tablets, capsules or soft gels it is possible to obtain gastro-resistant pharmaceutical forms. Once reached the intestine, the chemical compounds contained in the OEs are assimilated more or less quickly, with different bioavailability, depending on their chemical structure and pharmacokinetics. Geraniol rapidly crosses the enterocytes and, once reaching the bloodstream, has shown a half-life of 12.5 min with a pseudo first-order kinetics [5]. On the contrary, Eugenol is rapidly absorbed, but has shown a half-life of 18.3 h in blood and could accumulate after repeated daily administrations [123].

Cinnamaldehyde has shown a half-life of 6.7 h. However, the 60% of cinnamaldehyde is oxidized to cinnamic acid rapidly [124]. Carvacrol is only partially absorbed in the intestine and has shown to reach low concentration in plasma with a peak after 2 h of oral administration [125].

d-limonene is only partially absorbed by enterocytes and has shown a half-life of 3.20 h in plasma [126,127]. Similar behavior has been found for β-caryophyllene, that has shown a half-life of 4.07 h in plasma [128]. Thymol is absorbed quickly in the gut, and it is present in plasma only as thymol sulfate, that reach a considerable concentration after 20 min, with an half-life of 10.2 h [129].

The current scientific knowledges regarding their activities against oxidative stress and their ability to modulate the microbiota and the intestinal inflammation are more than enough to plan clinical trials on humans and provide evidences for their use as therapeutic agents. The strong multitarget antitumor activity of some molecules present in EO make them potentially very effective therapeutic strategies against CRC, also in combination with chemotherapies already in use. However, the maximum potential of these compounds seems to be expressed in the prevention of CRC, inflammation and intestinal dysbiosis. Table 4 summarize the main effects of OEs divided according to their main activity in the intestine and with the indication of the individual compounds mainly responsible for the action.

A limit to their use is the need of controlled release systems that target these molecules in the intestine area where their action is required. In fact, all these compounds are absorbed in the small intestine, some of them very quickly, such as geraniol, while others with less efficient transport mechanisms, such as limonene [5,123]. Nevertheless, possibilities for a controlled release of these molecules already exist, and it has already been proven that they are effective in humans to obtain a controlled release [8]. Further in vivo and clinical studies are necessary to understand their bioavailability, pharmacokinetics and mechanism of actions. Unfortunately, the low cost that these EOs have on average, and their non-patentability, make them little or not at all interesting for pharmaceutical industries, making missing the sponsors for the clinical studies necessary to finally validate their therapeutic efficacy in many different intestinal pathologies of the large bowel, such as inflammation, colitis, dysbiosis and CRC.

## Figures and Tables

**Table 1:** Derived no effect level (DNEL) and No Observed Adverse Effect Level (NOAEL) for oral administration of major single components of EO.

EOs Major Single Components	DNEL	NOAEL
	for Oral Administartion (mg/kg bw/day)	
**Geraniol**	13.75	550
**Eugenol**	3	300
**Carvacrol**	0.0444	40
**d** **-limonene**	4.76	1000
**Cinnamaldehyde**	0.417	250
**Thymol**	8.3	667
**Eucalyptol**	600	600
**Menthol**	4.7	188

Data refers to studies in vivo on rodents, from https://echa.europa.eu/it evaluated in vitro and in vivo.

**Table 2:** Essential oils, their major components in descending order and their activities recognized in vitro and in vivo.

Essential Oil	Major Compounds and Structures	Major Activity
*Zanthoxylum bungeanum* pericarp EO	terpinen-4-ol 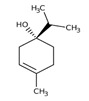	Anti-inflammatory
	eucalyptol 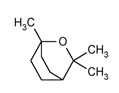	
	xanthoxylin 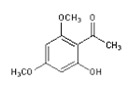	
*Zanthoxylum myriacanthum* EO	limonene 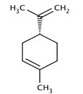	Anti-inflammatory; Microbiota modulation; Fungistatic;
	β-phellandrene 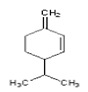	
	α-phellandrene 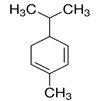	
*Citrus sinensis* EO	limonene 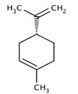	Anti-inflammatory; Microbiota modulation; Fungistatic
*Foeniculum vulgare* EO	trans-anethole 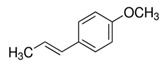	Anti-inflammatory;
*Zingiber officinale* EO	zingiberene 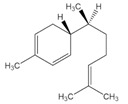	Anti-oxidant; Microbiota modulation;
	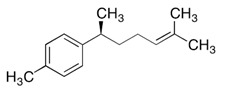 α-curcumene	
	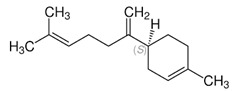 β-Bisabolene	
	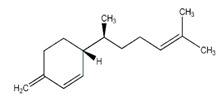 α-sesquiphellandrene	
*Ocimum basilicum* EO	linalool 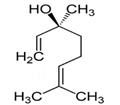	Anti-oxidant; Antiviral;
	β-pinene 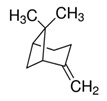	
	trans-verbenol 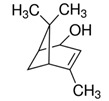	
	α-terpinolene 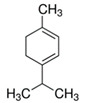	
*Oenothera biennis*, *Rosa chinensis, Helichrysum italicum and Cymbopogon citratus* EOs	Geraniol 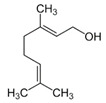	Anti-oxidant; Bactericide;
*Thymus vulgaris* EO	thymol 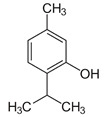	Anti-oxidant, Bactericide, Bacteriostatic, Microbiota modulation; Fungicidal; Anti-viral;
*Curcuma longa* EO	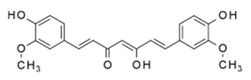 curcumin	Anti-inflammatory
	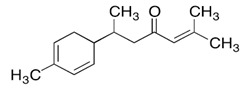 α-turmerone	
*Rosmarinus officinalis* and *Eucalyptus* spp. EO	eucalyptol 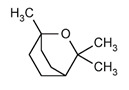	Bactericide; Antiviral;
*Origanum vulgare* EO	carvacrol (c) 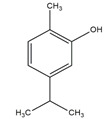	Anti-oxidant; Bactéricide, Microbiota modulation Antiviral
*Syzygium aromaticum* EO	eugenol 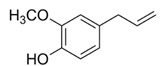	Bactericide; Biofilm inhibition; Fungicidal,
*Cinnamomum* spp. EO	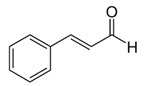 cinnamaldehyde	Bacteriostatic, Microbiota modulation; Fungicidal;
*Mentha spp.* EO	menthol 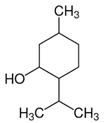	Bactericide Fungistatic

**Table 3:** Efficacy of different EOs in improving clinical and histological signs of animal models of colitis.

Essential Oil	Main Active Components	Tested Doses	Effect
*Zanthoxylum bungeanum*	Terpinen-4-ol, eucalyptol, xanthoxylin	20, 40 and 80 mg/kg body weight/day Effective dose required: all, but 80 mg/kg is most effective	DSS model. Reduced weight loss, DAI and histological damages, proinflammatory cytokines expression (TNF-α, IL-1 β and IL-12) induced by colitis. Decreased NF-kβ levels and increased expression of PPARγ in the colon wall (in vivo) [10]
*Zanthoxylum myriacanthum*	Limonene, β-phellandrene, α-phellandrene, α-pinene and o-cymene	35 and 70 mg/kg body weight/day Effective dose required: both doses are effective	DSS model. Similar then *Z. bungeanum* [11]
*Foeniculum vulgare*	Trans-anethole, fenchone, methyl chavicole and limonene	100, 200 and 400 mg/kg body weight/day Effective dose required: 200 and 400 mg/kg	Acetic Acid model. Reduced histological lesions induced by colitis and expression in mucosal mRNA levels of MPO, TNF-α and NF-kβ (in vivo) [12]
*Zingiber officinale*	Zingiberene, α-curcumene, β-Bisabolene and α-sesquiphellandrene	100, 200 and 400 mg/kg body weight/day Effective dose required: dose-dependent manner	Acetic Acid model. Reduced the extent of ulcerated areas, immune cell infiltrations and crypt damages induced by colitis (in vivo) [13,14]
*Ocimum basilicum*	Linalool, β-pinene, trans-verbenol and α-terpinolene	160 and 320 mg/kg body weight/day Effective dose required: both doses are effective	Acetic Acid model. Decreased of histological damages induced by colitis and MPO activity (in vivo) [15]
*Cymbopogon martini*	Geraniol	30 and 120 mg/kg body weight/day Effective dose required: 120 mg/kg but some effects are detected at lower dose	DSS model. Decreased DAI score, circulating TNF-α, IL-1 β, Il-17, IFNγ and COX-2 mRNA expression. Prevent weight loss, histological damages and dysbiosis induced by colitis (in vivo) [7]
*Curcuma longa*	Curcumin	25–50 mg /kg body weight/day Effective dose required: both doses are effective	DSS model. Anti-inflammatory cytokines including IL-10 and IL-11 as well as FOXP3 were upregulated [17]

TNF-α, Tumor Necrosis Factor-alpha; NF-kβ, Nuclear Factor kappa-light-chain-enhancer of activated B cells; DSS, Dextran Sulphate Sodium; DAI, Disease Activity Index; MPO, Myeloperoxydase; COX-2, Cyclooxygenase-2; FOXP3, Forkhead box P3.

**Table 4:** Effects of different essential oils on the intestine: molecules mainly involved and effects observed in vitro and in vivo.

**Antinflammatory Activity**				
**Essential Oil**	**Main Components**	**Effects**	**Mechanism of Action**	**References**
*Zanthoxylum bungeanum* (pericarp)	Terpinen-4-ol, eucalyptol, xanthoxylin	Reduction of: weight loss, DAI, histological damages, tissue TNF-α, IL-1 β and IL-12 raise induced by colitis (in vivo)	Decrease of NF-kβ and increase PPARγ expression	[10]
*Zanthoxylum myriacanthum*	Limonene, β-phellandrene αphellandrene	Reduction of: weight loss, DAI, histological damages, tissue TNF-α, IL-1 β, IL-6 and IL-12 p35 raise induced by colitis (in vivo)	Inhibition of phosphorylation of IKK and IκB	[11]
*Foeniculum vulgare*	Trans-anethole, fenchone, methyl chavicole, limonene	Reduction of the histological lesions induced by colitis (in vivo)	Decrease of MPO, TNF-α and NF-kβ expression	[12]
*Zingiber officinale* (Ginger)	Zingiberene, α-curcumene, β-Bisabolene, α-sesquiphellandrene	prevent colonic tissue damages Induced by colitis (in vivo)	Not specified	[14]
*Ocimum basilicum* (Basil)	Linalool, β-pinene, trans-verbenol, α-terpinolene	Decrease of histological damages Induced by colitis (in vivo)	decrease of the MPO activity	[15]
N.A.	Geraniol	decrease of DAI score, preventing weight loss and histological damages and dysbiosis induced by colitis (in vivo)	Regulation of Wnt/β-catenin, p38MAPK, NFκB, PPARγ and COX-2 signaling pathways	[7,19]
N.A.	Borneol, camphene and eucalyptol	N.A.	iNOS and COX-2 regulation	[18]
**Antioxidant Activity**				
**Essential Oil**	**Main Components**	**Effects**	**Mechanism of Action**	**References**
N.A.	Geraniol	protection from DNA methylation damages (in vitro), reduction of colon inflammation and lipid peroxidation (in vivo)	Decrease of iNOS activity, increase of antioxidant enzymes	[7,20,21,22]
*Zingiber officinale* (Ginger)	Not specified	reduction of intestinal lipid peroxidation, in a model of induce free radicals neutralization (in vivo)	increase of antioxidant enzymes and serum glutathione levels	[130]
N.A.	Carvacrol	Reduced colonocyte damages caused by ROS (in vivo)	Not specified	[131]
N.A.	Thymol	protection against oxidative DNA damage (in vitro)	Not specified	[121]
*Ocimum basilicum* (Basil)	Not specified	Anti-inflammatory (in vivo)	Reduction of MPO activity	[15]
**Antibacterial Activity and Microbiota Modulation**				
**Essential Oil**	**Main Components**	**Effects**	**Mechanism of Action**	**References**
N.A.	Geraniol	Antibacterial against *E. aerogenes, E. coli, P. aeruginosa* and *A. baumannii* (in vitro), microbiota modulation (in vivo), boosting of beneficial bacteria (*Collinsella* and *Faecalibacterium*)	destabilization of phatogen bacterial cell wall and damage of transmembrane efflux pumps	[7,8,29]
N.A.	Eugenol	Antibacterial against *E. coli*, *L. monocytogenes* and *L. sakei* (in vitro), *C. rodentium, P. aeruginosa* and *E. coli* (in vivo)	Permeabilization of the bacterial membrane, depletion of ATP, DNA damage, inhibition of the intestinal bacterial adherence, inhibition of quorum sensing	[31,32,33,34]
N.A.	Cinnamaldehyde	Bacteriostatic against *S. aureus, E. cloacae, A. baumannii* and *L. monocytogenes,* bactericidal against *E. coli, E. faecalis, E. faecium, E. aerogenes S. enterica* and *C. perfringens, C. difficile* (in vitro), improvement of the ecological biodiversity (in vivo)	inactivation of its acetyl-CoA carboxylase enzyme	[35,36,37,38,39]
N.A.	Thymol	Bacteriostatic against *C. difficile, C. perfringens, P. shermanii, P. freudenreichii and B. thetaiotaomicron* (in vitro) Bactericide against *L. monocytogenes, E. coli, S. enterica, S. aureus, C. botulinum, C. perfringens, S. sonnei, S. lutea, M. flavus, B. hermosphacta, L. innocua, L. monocytogenes, P. putida and S. putrefaciens* (in vitro), Modulation of intestinal microbiota (in vivo)	Disruption of bacterial biofilm	[26,30,41,42,44,45]
N.A.	Carvacrol	Bactericide against *E. coli*, *Salmonella*, *Campylobacter* spp. (in vivo) Microbiota modulation	Inhibition of bacterial adhesion, invasion and biofilm development	[46,47]
N.A.	Limonene	Microbiota modulation	Not specified	[50]
*Eucalyptus*	Eucalyptol	*Bactericide against S. aureus*, *E. coli, B. subtilis, K. pneumonia, S. enteritidis* and *P. aeruginosa*. (in vitro)	Destabilizing and disrupting bacteria membrane	[52]
N.A.	Menthol	*C.difficile* decrease (in vitro)	Barrier disruption and significant leakage of cellular ATP	[54]
**Antimycotic Activity**				
**Essential Oil**	**Main Components**	**Effects**	**Mechanism of Action**	**References**
*Thymus vulgaris, Citrus limonum, Pelargonium graveolens, Cinnamomum cassia, Ocimum basilicum, and Eugenia caryophyllus*	Not specified	Fungistatic and Fungicidal against *C. albicans* and *C. glabrata* (in vitro)	Not specified	[64]
N.A.	cinnamaldehyde	Inhibition of *C.albicans* growth (in vitro)	Damage of cell membranes, modulation of potassium ion efflux	[65]
N.A.	Limonene	Fungistatic on *C.albicans* (in vitro)	Inhibition of adhesion, development and maturation of biofilm	[66,67]
Mentha	Menthol and (+)-carvone	Fungistatic on *C.albicans* (in vitro)	Not specified	[68]
Caraway	Not specified	Fungistatic on *C.albicans* (in vitro)	Not specified	[69]
*Thymus vulgaris*	Thymol, carvacrol	antifungal activity against *C.albicans, Aspergillus, C.tropicalis* and dermatophyte species (in vitro)	Damage of cell membranes	[27,70,71,72]
Clove oil	Eugenol	biofilm inhibition of *C. albicans* (in vitro)	Damage to cell membranes, ATP depletion	[27,73,74,75,76,77]
		**Antiviral Activity**		
**Essential Oil**	**Main Components**	**Effects**	**Mechanism of Action**	**References**
N.A.	camphor, thymol and eucalyptol	Decreased infectivity of Bovine viral diarrhea virus	Damage of viral particle	[82]
N.A.	carvacrol	Reduced propagation of nonenveloped murine norovirus	Damage of viral capsid and RNA	[83]
**Anticancer Activity**				
**Essential Oil**	**Main Components**	**Effects**	**Mechanism of Action**	**References**
N.A.	Carvacrol	Antiproliferative effect (in vitro)	Block of cell cycle in G2/M phase, reduction of invasion and migration (HTC-116 and LoVo)	[105]
N.A.	Geraniol	Cytotoxic effect (in vitro) Cytostatic effect (in vitro) Regualtion polyamine metabolism (in vitro) Genoprotective effect (in vitro) Antitumoral effect in association with chemotherapeutic agents (in vitro and in vivo)	Induction of apoptosis (Colo-205); Block of cell cycle in S phase, downregulation of ODC and AdoMetDC, Reduction of methylation and ROS DNA damage, sensitization of cells to 5-FU; Inibithion of cell growth by downregulating TS and TK (SW-620)	[106,107,108,113,114]
N.A.	Thymol	Cytotoxic effect (in vitro) Genoprotective effect (in vitro)	Induction of ROS production and DNA damages, induction of cell death by mitochondrial pathways (HTC-116); Reduction of methylation and ROS DNA damage	[21,109]
N.A.	Cinnamaldehyde	Antiproliferative effect (in vitro) Antitumoral effect in association with chemotherapeutic agents (in vitro and in vivo)	Induction of apoptosis by an increase of Bax/Bcl-2 ratio, inhibition of proliferation by PI3K/Akt pathways, reduction of invasion and migration by increasing E-cadherin levels and downregulation of MMP-2 and MMP-9 (SW-480). Induction of apoptosis in association with camptothecin	[110,111,112]
N.A.	Thymoquinone	Antitumoral effect in association with chemotherapeutic agents (in vitro)	Increasing antineoplastic effect of doxorubicin, Increasing cell death by suppressing NF-kβ in association with cisplatin, Induction of autophagy in association with irinotecan	[116,117,118,119]
N.A.	β-caryophyllene	Antitumoral effect in association with chemotherapeutic agents, regulation of glucose homeostatis (in vitro)	Increase of anticancer activity of paclitaxel and doxorubicin Regulation of genes involved in glycolysis and cell growth, induction of apoptosis	[119,120,121]

## Abbreviations

ATP	Adenosine triphosphate
COX-2	Cyclooxygenase-2
CRC	Colorectal Cancer
DAI	Disease Activity Index
DSS	Dextran Sulfate Sodium
EO	Essential Oil
FAP	Familial Adenomatous Polyposis
FOXP3	Forkhead box P3
GALT	Gut-Associated Lymphoid Tissue
HFD	High Fat Diet
IBS	Irritable Bowel Syndrome
IBD	Inflammatory Bowel Disease
MIC	Minimum Inhibitory Concentration
MPO	Myeloperoxidase
NF-kβ	Nuclear Factor kappa-light-chain-enhancer of activated B cells
NOS	Nitric Oxide Synthase
ROS	Reactive Oxygen Species
TNF-α	Tumor Necrosis Factor-alpha
